# Effect of various different pretreatment methods on infrared combined hot air impingement drying behavior and physicochemical properties of strawberry slices

**DOI:** 10.1016/j.fochx.2024.101299

**Published:** 2024-03-20

**Authors:** Da-Long Jiang, Qing-Hui Wang, Chu Huang, Parag Prakash Sutar, Ya-Wen Lin, Samuel Ariyo Okaiyeto, Zi-Fan Lin, Yun-Tian Wu, Wen-Ming Ma, Hong-Wei Xiao

**Affiliations:** a School of Computer and Control Engineering, Yantai University, Yantai 264005, Shandong, China; b Agricultural Mechanization Institute, Xinjiang Academy of Agricultural Sciences, Urumqi, China; c College of Engineering, China Agricultural University, P.O. Box 194, 17 Qinghua Donglu, Beijing 100083, China; d Yancheng Dafeng District Fruit Tree Technical Guidance Station, Yancheng 224005, Jiangsu, China; e Department of Food Process Engineering, National Institute of Technology Rourkela, Odisha 769008, India; f School of Food Science and Engineering, Bohai University, Jinzhou 121000, Liaoning, China; g Department of Electrical and Electronic Engineering, University of Western Australia, Perth 6000, Australia; h BeiGene Guangzhou Biologics Manufacturing Co., Ltd, Guangzhou 510555, China

**Keywords:** Freezing combined ultrasound pretreatment, Strawberry slices, Water state, Ultrastructure, Antioxidant activity, Volatile compounds

## Abstract

In current work, the effect of freezing (F), ultrasound (U), and freeze- ultrasound (FU) pretreatment on infrared combined with hot air impingement drying kinetics, cell ultrastructure, enzyme activity, and physicochemical properties of strawberry slices were explored. Results showed that FU pretreatment enhanced cell membrane permeability via forming micropores, altered water status by transforming bound water into free water and thus promoted moisture diffusivity and decreased drying time by 50% compared to the control group. FU pretreatment also extensively decreased pectin methylesterase enzyme activity and maintained quality. The contents of total phenols, anthocyanins, vitamin C, antioxidant activity, and a* value of dried strawberries pretreated by FU were extensively increased compared to the control group. U and FU pretreatments were beneficial for retaining aromatic components and organic sulfides according to e-nose analyses. The findings indicate that FU is a promising pretreatment technique as it enhances drying process and quality of strawberry slices.

## Highlights

• Drying time of freeze- ultrasound pretreatment (FU) group was significantly reduced by 50% compared to control group.
• FU pretreatment resulted in better microstructure of strawberry slices and promotes water redistribution.
• FU pretreatment effectively maintained volatile aroma of strawberry slices.
• The dried products pretreated by FU had better color, texture and nutritional content.

## Introduction

Strawberries are popularly consumed due to their sweet taste, aromatic flavour, and rich nutrients like polyphenols, anthocyanins and vitamin C, making them one of the most popular fruits in international commercial trade (Wang et al., 2022). At present, China (3.39 × 10^6^ t), the United States (1.21 × 10^6^ t), Egypt (4.71 × 10^5^ t), Mexico (5.43 × 10^5^ t), and Turkey (6.69 × 10^5^ t) are the top five strawberry producers worldwide, accounting for 68.52 % of the world’s strawberry production annually (FAO, 2024). However, strawberries are very susceptible to spoilage after harvest and are unsuited for long storage and transportation as they are prone to tissue softening, spoilage and browning. The probability of postharvest rot of strawberry fruits is about 40 % (Ren, Yang, Feng, He, & Huang, 2023), which essentially limits the shelf life of strawberries and their commercialization and causes substantial economic losses.

Drying is the most common techniques of fruit preservation as it can extend the shelf life, minimize microbial activity, prevent biochemical, chemical, and physical deterioration, and reduce transportation and storage costs (Xiao & Mujumdar, 2020). At present, freeze-drying is often employed to produce strawberry chips, which ensures maintenance of the shape, colour, taste, aroma, and nutritional composition of the dried products to the greatest extent (Zhang et al., 2020, Zhang et al., 2024). Nevertheless, freeze-drying is undoubtedly expensive and energy-consuming. Considering the successful application of infrared drying in pineapple slices (Malakar, Arora, Nema, & Yadav, 2023), *Stevia rebaudiana* leaves (Ai et al., 2022), etc., some researchers also use infrared radiation combined with other drying technologies to dry strawberry chips (Adak, Heybeli, & Ertekin, 2017). Unfortunately, the limited penetration of infrared radiation and the higher sugar content in strawberries results in longer drying times and more energy consumption (Adak et al., 2017). The use of pretreatment before drying could accelerate moisture migration and diffusion during the drying process, improve energy utilization and drying efficiency (Deng et al., 2019, Wang et al., 2019). In consequence, it is very attractive to explore suitable pre-treatment methods and conditions to preserve the nutrients and colour of strawberries, as well as reduce drying time and improve energy efficiency. Some scholars (Hu et al., 2022, Sun et al., 2024, Yang et al., 2023) further improved the chewability, colour and other sensory qualities of freeze-dried strawberries by adding edible gum, pectin and gradually expanded the strawberry pulp processing industry. But food glue is difficult to digest in the human stomach, and excessive consumption would cause damage to the gastric mucosa (He, Bian, Xie, & Chen, 2021).

Recently, ultrasonic technology is often used as a pre-treatment method before drying because it can make the material continue to shrink and expand, forming a sponge-like structure by damaging the cell wall and formation of micro-channels. Ultrasound-assisted drying improves moisture removal rates and product quality such as blackberries (Tao et al., 2021), scallion stalk slices (Zhou et al., 2022), garlic slices (Tao et al., 2018), etc. Wang et al. (2019) also obtained negative results that ultrasonic pretreatment reduced the ascorbic acid and solid contents of kiwi fruit slices. Therefore, the results of different ultrasonic treatment conditions for different materials are not necessary.

Freezing pre-treatment is another effective alternative to improve the drying rate because the formation of ice crystals could change the permeability of cell membranes and destroy the structure of cell walls. The drying time (37.55 %) and energy consumption (36.64 %) of *Cistanche deserticola* treated with freeze–thaw were significantly shorter than those in untreated group (Ai et al., 2023). Taking into account the many advantages of the above two pretreatment methods, Liu, Wang, Lv, Li, and Wang (2021) combined freezing and ultrasonic pretreatment (FU) for drying the medicinal plant (*Platycodon grandiflorum* slices). Drying results indicated that the FU pretreatment method could not only preserve the content active compounds (*Platycodin D*) and aroma components well, but also improve the drying rate. However, the investigation of combining the two pretreatment techniques on the drying performance of fruit and vegetable materials are less studied, especially the application before the drying of strawberry slices has not been reported in detail. What are the effects of combined pretreatment on total phenols, anthocyanins, vitamin C, antioxidant activities and other nutrients of strawberry slices after infrared drying? The effects of combined pretreatment on the content and distribution of free water and bound water in strawberry slices are still unclear. The related mechanisms of ultracellular structure, texture and water status on drying kinetics have not yet been explained.

Therefore, the purpose of this work is to comparatively analyse the effects of various pretreatment techniques F, U, and FU on the drying kinetics and physicochemical quality of strawberries after infrared combined hot air impingement drying (IHAID). By characterizing the effects of various pretreatment methods on the texture, ultrastructure and moisture status of strawberry slices, the influencing mechanisms behind the phenomenon were explored.

## Materials and methods

### Materials

Fresh strawberries (Dandong 99) were sourced in a strawberry greenhouse (Jinzhou, Liaoning, China) in May 2023. Ripe and intact strawberries with uniform shape (longitudinal diameter of (44.0 ± 0.8) mm and equatorial diameter of (35.3 ± 0.9) mm), mass (21.2 ± 1.7) g, and colour were selected for the experiments. The strawberries were cleaned and the middle of the strawberries were selected. The selected part was cut into circular slices with a thickness close to about 5.0 mm. The wet basis moisture content of raw samples amounted to (89.15 ± 0.20)%, which was measured by vacuum low-temperature drying until the mass stabilizes.

### Pretreatment experiments

**Fig. 1:**
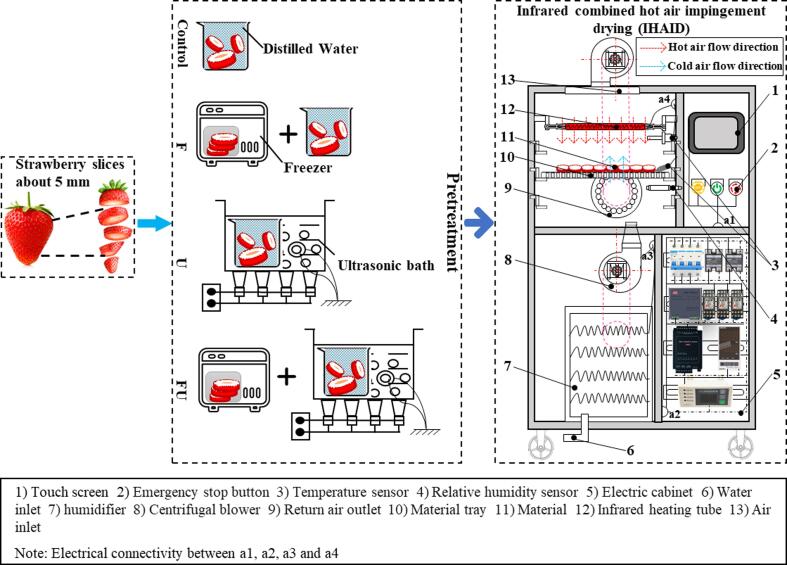
Schematic diagram of drying strawberry slices by IHAID dryer under various pretreatment methods.

As shown in Fig. 1, the middle part of the washed strawberries was cut into uniformly sized discs and evenly divided into four equal parts (about 500 g each). After pre-experiments, 16 min was determined as the unified processing time for different pretreatment methods. The strawberry slices in the control group were immersed in deionized water for 16 min at room temperature. The strawberry slices of group F were frozen in a freezer (BL-200WS1580L, Shenzhen Yingpeng Electric Appliance Co., LTD, GuangDong, China) at −20 °C for 20 h, then taken out and soaked in deionized water for 16 min. The strawberry slices of group U were placed in an ultrasonic instrument (TS-6000 single slot ultrasonic cleaning machine, Jinan Taishan Electric Appliance Factory Co. LTD, Shan Dong, China.) for 16 min of the parameters at 30 kHz and 180 W. The strawberry slices in the FU group, that is, the combination of the F and U groups, were first frozen for 20 h and then ultrasonicated for 16 min. All pretreatments were performed in six replicates.

### IHAID dryer

This dryer for this experiment was developed at the College of Food Science and Engineering, Bohai University, Jinzhou City, Liaoning Province, China. The dryer mainly includes heating, humidification, control and air circulation system, as shown in Fig. 1. The control system determined whether to run the ultrasonic humidifier according to the comparison between drying room medium relative humidity and the value set on the touch screen. Radiant power, wavelength, and other component installation details are described in detail in the articles by Zhang, Zhu, et al. (2020) and Ai et al. (2022).

A radiation temperature at 60 °C, radiation power at 950 W, radiation distance at 10 cm, and pretreatment time of 16 min were chosen as the parameters of the subsequent experiment based on the preliminary experiments. Periodically measure the sample mass during drying until the material moisture is below 0.1 (dry basis), thus achieving the moisture content value for long-term safe storage.

### Analytical methods

#### Moisture ratio measurement

The moisture ratio of the strawberry slice was usually calculated by the simplified Eq. (1) (Wang et al., 2019):

(1) $$\mathrm{MR}=M_{t}\text{/}M_{0}$$

where *MR* expresses the moisture ratio of the sample, *M*_0_ expresses the initial dry base moisture content (g/g), *M*_t_ expresses the dry base moisture content (g/g) at time *t* during drying.

#### Colour measurement

According to the method described by Zhang, Ha, et al. (2024), the Δ*E* was calculated according to Eq. (2):

(2) $$\Delta E=\sqrt{{\left({\text{L}}_{0}-L*\right)}^{2}+{\left({\text{a}}_{0}-a*\right)}^{2}+{\left({\text{b}}_{0}-b*\right)}^{2}}$$

where Δ*E* expresses the total colour change; L*, a*, b* and *L*_0_, *a*_0_, *b*_0_ express the brightness/darkness, redness/greenness, yellowness/blueness of the strawberry slices after drying and fresh strawberry slices, respectively.

#### Microstructure analysis

According to method of Zhou et al. (2022), the dried strawberry slices of different pretreatment groups were photographed (×300 magnification) by scanning electron microscopy (SU3500, Hitachi Co., Tokyo, Japan) of the accelerated voltage at 15 kV.

#### Determination of volatile composition

Electronic nose (PEN3; Air sense, Schwerin, Germany) analysis of volatile compounds in strawberry slices was implemented according to Xu et al. (2021). 2 g of dried strawberry powder from different pretreatment groups was put into a 20 mL vial, and placed in a water bath. The sampling injection flow capacity was set to 400 mL/min, and the measurement was repeated three times. Differences among samples were analyzed by principal component analysis (PCA).

#### Determination of nutrient content in strawberry slices

Strawberry extract was obtained referring to the detail described by Xu et al. (2021). Two grams of strawberry powder was weighed and mixed with 20 mL of 70 % ethanol solution (V/V), then ultrasonically extracted (SY-360 Ultrasound Cleaner, Weimi Technology Co., Ltd., Shanghai, China) for 30 min, centrifuged (TG20G centrifuge, Spring Instrument Co., Ltd, Jiangsu, China) for 15 min, and the supernatant was collected. The collection was repeated in triplicate.

##### Measurement of total anthocyanin content (TAC)

TAC content was determined with reference to the method mentioned by Kanha, Regenstein, Surawang, Pitchakarn, and Laokuldilok (2021). The TAC was expressed as anthocyanin 3-glucoside (C3G) mg/g dry mass. For all experiments, six replications were performed (n = 6).

##### Measurement of total phenolic content (TPC)

TPC was measured by referring to the methods of Yao, Fan, and Duan (2020). The TPC was presented as gallic acid equivalent (GAE, mg/g DW). For all experiments, six replications were performed (n = 6).

##### Determination of vitamin C (VC)

Referring to the modified method of Sakooei-Vayghan, Peighambardoust, Hesari, and Peressini (2020), the content of vitamin C in dried strawberry slices (mg/100 g d.m.) was determined by titration with 2,6-dichlorophenol-indoxyl (DCPIP). All experiments were repeated six times (n = 6).

#### Determination of antioxidant capacity of strawberry

According to the method mentioned by Xu et al. (2021), DPPH and hydroxyl radical scavenging ability of strawberry samples were measured.

#### Analysis of low-field nuclear magnetic resonance detection

The program parameters established by Xu et al. (2021) and Zhang, Qiao, et al. (2020) were used to measure the water distribution of different pre-treated and dried sample by a low-field nuclear magnetic analyzer (Niumag Co., Ltd., Shanghai, China).

#### Analysis of texture properties of strawberry

The procedure mentioned by Ai et al. (2022) was used to measure the effect of various pretreatment techniques on the texture characteristics of strawberry slices. Measurements were repeated six times for each group.

### Statistical analysis

The experimental value was statistically analyzed by SPSS software v.20.0 (IBM Corp., Armonk, NY, USA). Significant variation between groups were analyzed using ANOVA and the Duncan's test (*p* < 0.05).

## Results and discussion

### Moisture ratio

**Fig. 2:**
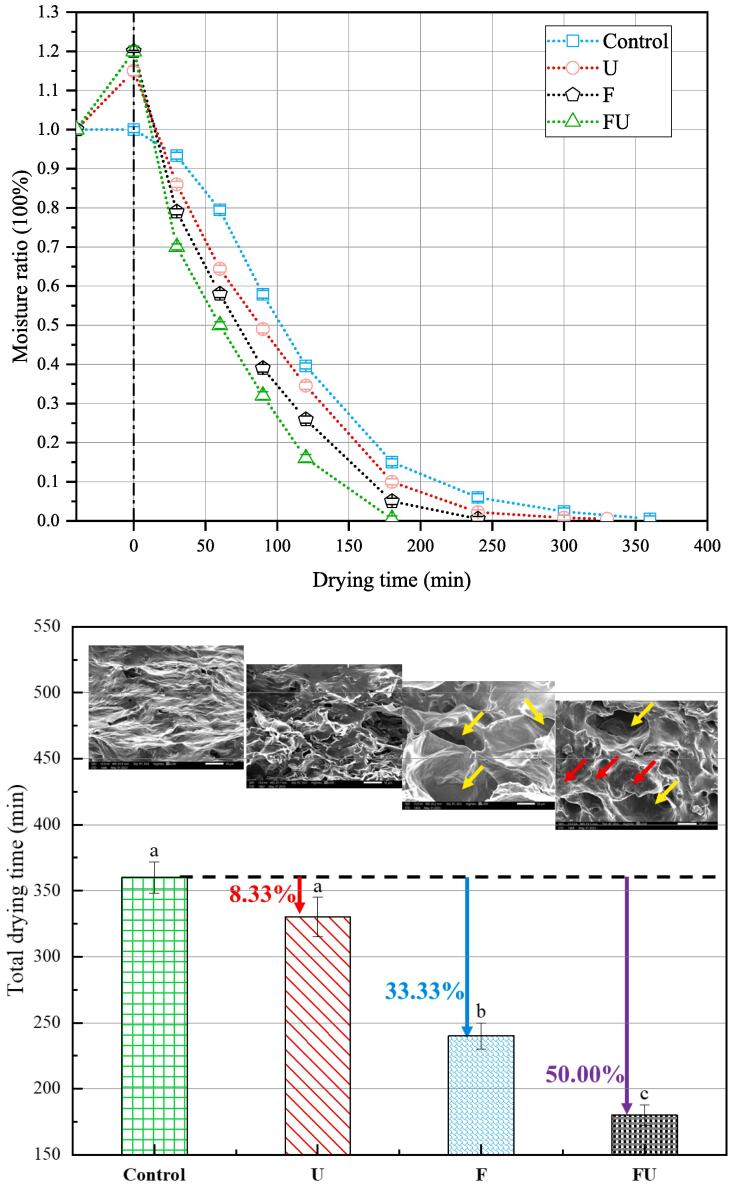
Effect of various pretreatments on moisture ratio and microstructure. Magnification: 300×. Data in columns with the different letters (a–c) are significantly different (*p* < 0.05). Control: untreated group; F: freeze–thaw pretreatment; U: ultrasound pretreatment; FU: freeze- ultrasound pretreatment.

IHAID was used to dry strawberry slices after different pretreatment methods to analyze the effects of various pretreatments on their drying behavior (Fig. 2). The initial moisture content of the F, U, and FU samples increased slightly before drying because the destruction of cell structure after pretreatment results in deionized water penetrating into the cells. The total drying time of F, U, and FU samples was reduced by 33.33 %, 8.33 %, and 50.00 % compared to the untreated samples, respectively. Besides, the moisture content of the pretreated (F, U, and FU) samples decreased faster than the control sample.

Ultrasonic pretreatment (U) before drying was significant (*p* < 0.05) and increased the drying rate of strawberry slices due to the generation of ultrasonic microchannels in the internal components of strawberries. Xu et al. (2021) reported that sequential dual-frequency mode (SeDM) ultrasonic treatment of strawberries significantly reduced the drying time by 50 % compared with the untreated group. Similar results were observed from other products including jackfruit (Wu, Guo, Guo, Ma, & Zhou, 2021), and sweet potato (Oladejo, Ma, Qu, Zhou, & Wu, 2017).

Freezing pretreatment (F) could damage the cell membrane structure of strawberries, and accelerate their drying speed. Ai et al. (2023) reported similar results that freeze–thaw pretreatment significantly altered the cellular structure, functional properties, water status and distribution of *Cistanche deserticola*, as well as significantly reduced drying time. Ando et al. (2019) showed that freezing pretreatment significantly reduced drying time of carrots by destroying the membrane structure and changing the main components of the cell wall to enhance water diffusion.

The highest drying rate of the samples was observed after FU pretreatment, which may be caused by the combined effect of freeze–thaw treatment to enhance cell permeability and ultrasonic treatment to form water molecules cavitation in the solid matrix (Liu et al., 2021).

### Changes in colour of dried samples

**Fig. 3:**
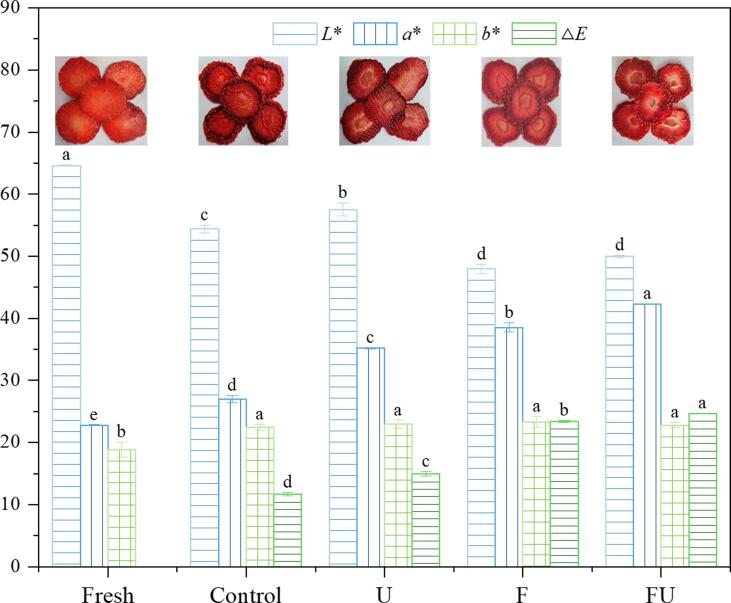
Effect of various pretreatments on the colour of IHAID dried strawberry slices. Control: untreated group; F: freeze–thaw pretreatment; U: ultrasound pretreatment; FU: freeze- ultrasound pretreatment.

Colour of strawberry slices is the major influences on the acceptance of consumers because it is the most intuitive to feel. The L* value of sliced strawberries after IHAID was significantly lower than that of the fresh strawberries, while the a* value was significantly higher (Fig. 3). Shrinkage deformation and structural damage in drying may absorb large amounts of brightness and divert photons, resulting in lower L* value (Sun et al., 2022, Zhou et al., 2022). Besides, the L* value of dry samples in the U group was significantly higher than that in the untreated group, and the L* value in the F group was the lowest. After drying, the b* value of strawberry slices did not change significantly, but the △*E* value of pretreated samples had a significant difference, and the FU group was the highest. This indicated that browning occurred in the drying process of IHAID to a certain extent. F treatment accelerated the browning reaction due to cell fluid outflow. In contrast, ultrasonic treatment reduced the oxygen content in strawberry samples and limited the browning reaction (Tao et al., 2021).

The a* value was the foremost reference indicators for strawberry slicing, and the a* value of the FU group was the highest, followed by F group and U group. This indicates that the strawberry cell fluid and anthocyanins were destroyed in the pre-treatment procedure, and they were unleashed into the cell space, which showed a stronger red colour at the surface of strawberries. In summary, FU pretreatment had the potential to produce ruddy and attractive dried strawberry slices, which may be related to the short drying time.

### Analysis of microstructure

The cross-section microstructure of samples is displayed in Fig. 2. The cells of the control sample were arranged in a smooth and dense manner, with cell collapse, compact structure, cell contraction and cell wall distortion. After U treatment, the porous structure was formed, the cell body was broken, and small cracks appeared at the cell body boundary. Xu et al. (2021) also found a similar phenomenon after treating strawberries under different ultrasonic frequency modes. During the freezing process, ice crystals formed inside the material, resulting in a high ion concentration gradient in and out of the samples, which will lead to irreversible stretching and fatal damage to the integrity of cell walls and cell membranes. Similar to the research results of Qiu et al. (2022), freezing treatment increased the cell wall gap of materials and formed a large number of irregular channels (Given by yellow arrows). Previous studies (Xu et al., 2021) have indicated that the formation of ice crystals contributes to the diffusion and evaporation of internal water, which also explains the differences in drying rates between different pretreatment methods in Section 3.1. Furthermore, FU group samples showed more significant irregular channels and a lot of small cracks (Given by red arrows), which was due to the effect of ultrasonic energy on cells expansion and contraction after freezing.

### Electronic nose

**Fig. 4:**
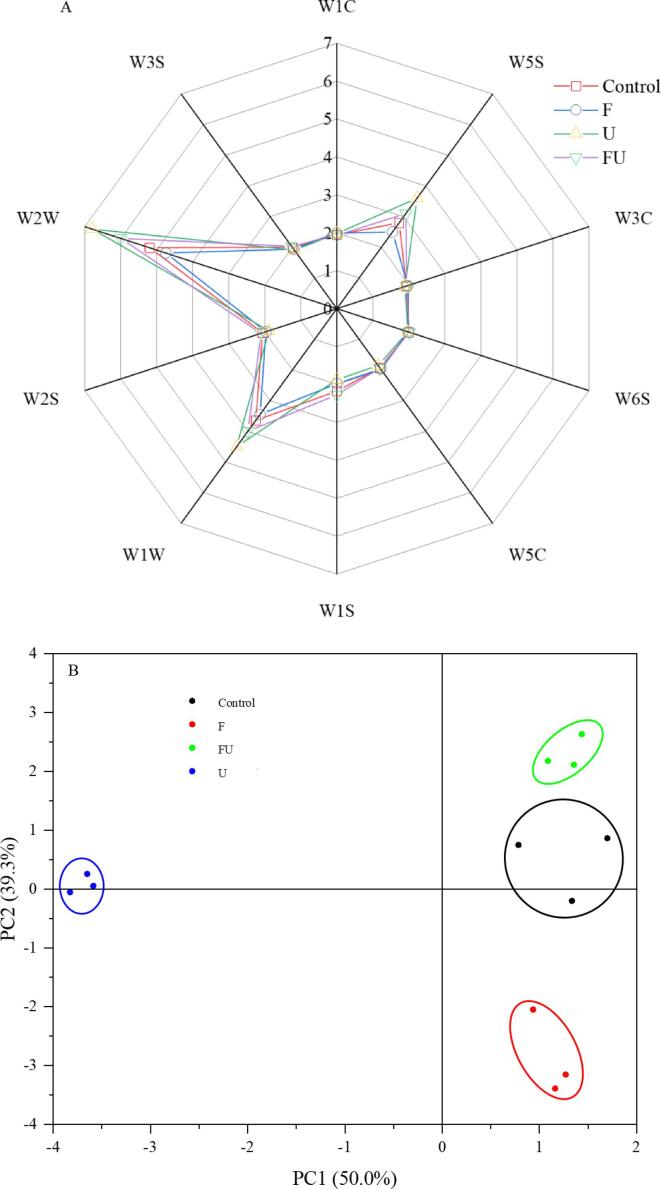
Effect of various pretreatment methods on flavour substances of strawberry slices. A: Radar chart; B: Principal component analysis. Control: untreated group; F: freeze–thaw pretreatment; U: ultrasound pretreatment; FU: freeze- ultrasound pretreatment.

In addition to colour, aroma is one of the key sensory indicators for consumers to directly measure the quality of dried strawberries. As shown in Fig. 4 (A), the response data of the four groups of samples were approximate, yet the signal intensity was a little dissimilar, among which W5S, W1W, and W2W were very significant. The larger the W2W value, the richer the aromatic compounds and the better the flavour of the dried strawberry. W1W and W5S stand for organic sulfides and nitrogen oxides, respectively, which have a negative effect on flavour. The U group samples had the highest W2W, W1W, and W5S response values, followed by the FU, the control, and the F groups in order. The W2W, W1W, and W5S values of F group samples were significantly lower than those of other groups. These results demonstrated that appropriate U pretreatment had a good effect on reducing the loss of volatile substances while freezing pretreatment would damage the cell membrane integrity and lead to the loss of volatile substances (Liu et al., 2021, Zhao et al., 2023).

The PCA plot (Fig. 4(B)) further reflects whether the E-nose data changes within the dried strawberry slices. The accumulated contribution rate of PC1 and PC2 major components was 89.3 %, indicating that the e-nose can better indicate the differences between samples from different pretreatment groups. FU, control and F groups were distributed on the right side, and U group was distributed on the left side, indicating that the first principal component is significantly different. Similarly, the FU, control, and U groups were located on the upper side, and the F group was distributed on the lower side, indicating that the second principal component had a significant difference. This trend corresponds with the radar chart results.

### Changes in chemical composition of dried samples under various pretreatments

Anthocyanins, total phenols, and vitamin C are the main bioactive components in strawberry fruits, and anthocyanins are also the main colorants for their red colour. From Table 1, different pretreatment methods significantly affected the bioactive components of strawberry slices (*p* < 0.05). The instability of anthocyanins is mainly influenced by chemical and physical factors such as light, heat, and oxidation during processing and storage (Cortez, Luna-Vital, Margulis, & Gonzalez De Mejia, 2017). The anthocyanin content was the highest in the FU group, followed by the F group, the U group, and the control group. There are three possible reasons for this result. Firstly, the operation time of the dehydration process after ultrasonic or freeze–thaw pretreatment was reduced (Fig. 2), and the heat exposure time of heat-sensitive substances was shortened. Secondly, ultrasound or freeze–thaw pretreatment damaged the cell structure (Fig. 2). Although it leads to the loss of a few heat-sensitive components including polysaccharides, polyphenols and flavonoids due to the destruction of cell structure to a certain extent, it also promotes the extraction of these components (Ai et al., 2023). Thirdly, ultrasonic treatment effectively reduced the oxygen content in strawberry cells and inhibited the activity of endogenous enzymes, thus reducing the degradation of strawberry anthocyanins.

**Table 1:** Nutrient content, antioxidant capacity and texture properties of strawberry samples under different pretreatment conditions.

Treatment	TAC (mg C3G/g)	TPC (mg GAE/g)	VC (mg AA/g)	DPPH (%)	–OH (%)	Hardness (N)	Chewiness (N)
Control	0.98 ± 0.01^c^	25.84 ± 0.11^c^	1.74 ± 0.06^c^	54.68 ± 0.27^c^	95.17 ± 0.14^c^	182.02 ± 0.63^a^	73.33 ± 0.94^a^
U	1.08 ± 0.02^b^	31.36 ± 0.12^b^	1.93 ± 0.01^bc^	62.69 ± 0.37^b^	103.24 ± 0.32^b^	86.33 ± 0.17^c^	31.24 ± 1.26^c^
F	1.12 ± 0.01^ab^	31.24 ± 0.06^b^	2.12 ± 0.08^b^	62.34 ± 0.52^b^	103.96 ± 0.76^b^	104.81 ± 2.25^b^	52.82 ± 0.88^b^
FU	1.19 ± 0.03^a^	33.87 ± 0.09^a^	2.44 ± 0.04^a^	65.51 ± 0.76^a^	108.32 ± 0.21^a^	51.68 ± 1.87^d^	20.69 ± 1.74^d^

Note: Numerical data are the means ± standard deviations of three quantities. Different letters in the same column (a, b, c, etc.) indicate significant differences (*P* < 0.05). Control: untreated group; F: freeze–thaw pretreatment; U: ultrasound pretreatment; FU: freeze-ultrasound pretreatment.

Vitamin C is unstable during food processing, easily decomposed by light, heat, and other factors, and is highly oxidized (Horuz, Bozkurt, Karataş, & Maskan, 2017). The vitamin C content in the control group was 10.9 %, 21.8 %, and 40.2 % lower than that in U, F and FU groups, respectively. This is mainly since the short drying time will reduce the loss of vitamin C.

Similarly, the total phenol content of the control, U, F, and FU groups was 25.84, 31.36, 31.24, and, 33.87 mg/g, respectively. Surprisingly, the difference in TPC between the U and F groups was not significant (*p* > 0.05). Although the drying time of F group was shorter than that of U group, the cavitation effect of the ultrasonic wave led to easy extraction of phenolic compounds or the passivation of phenolic degrading enzymes (Liu et al., 2022). Therefore, the addition of ultrasonic treatment before the drying process can contribute to the retention of bioactive ingredients.

Antioxidant activity was also one of the critical quality evaluation indexes of strawberry slices. The DPPH and –OH clearance rates of strawberry samples in the FU group were the highest, 65.51 % and 108.32 %, respectively. The DPPH content of the control group was 14.6 %, 14.0 %, and 19.8 % lower than that of the U, F, and FU groups, respectively. The changing trends of –OH, DPPH free radical scavenging abilities and the total phenolic content are basically similar. This phenomenon was consistent with previous research findings of previous studies (Liu et al., 2022), which showed a significant correlation between antioxidant capacity and phenolic content. In addition, ultrasonic treatment can increase hydroxyl content, improving antioxidant activity (Oladejo et al., 2017).

### Changes in moisture status of sample under various pretreatments

**Fig. 5:**
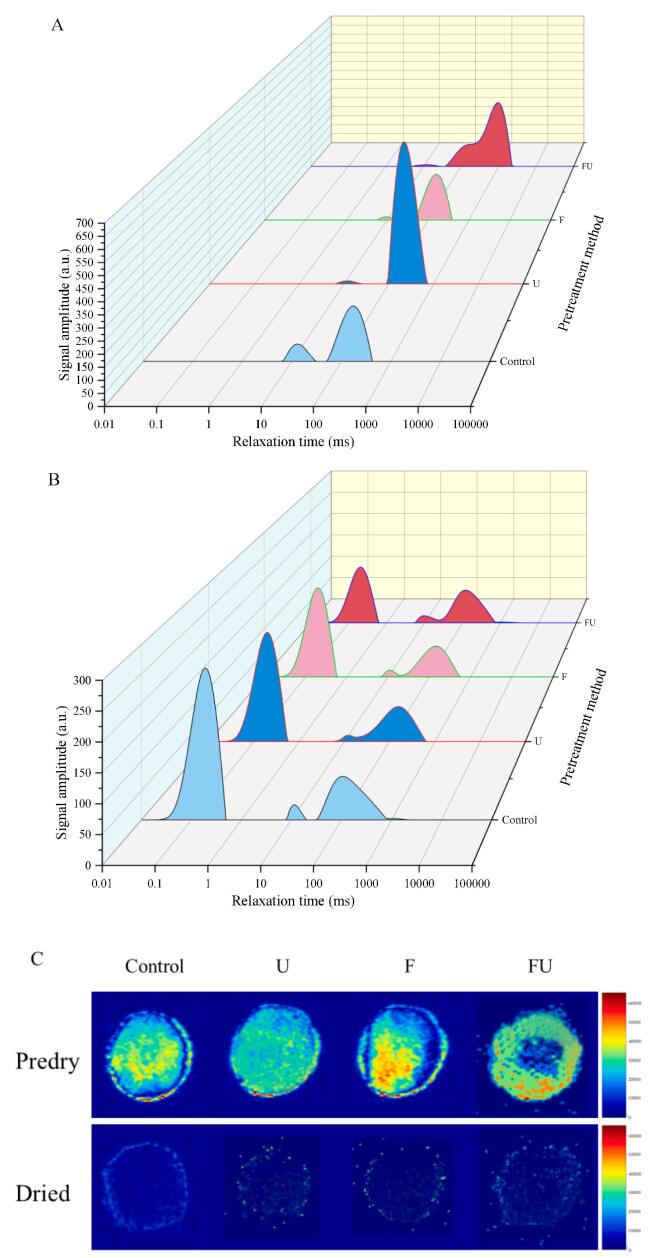
Typical distribution of T_2_ relaxation time (A and B) and MRI images (C) of various pretreatment methods on IHAID dried strawberry slices before and after drying. Control: untreated group; F: freeze–thaw pretreatment; U: ultrasound pretreatment; FU: freeze- ultrasound pretreatment.

The transverse relaxation time (T_2_) effectively represents the chemical environment of hydrogen protons and is commonly used to estimate the mobility of water. The T_2_ curve of sample showed three obvious peaks (Fig. 5(A)). The three peaks represent free water T_23_ (greater than 100 ms), semi bound water T_22_ (10–100 ms), and bound water T_21_ (0–10 ms), respectively. Similar findings were found in the procedure of freeze drying strawberry round slices after ultrasonic pretreatment by Xu et al. (2021).

Undried strawberries mainly contain free water and, a small amount of semi-bound water and bound water. Notably, the semi-bound water and bound water content of the samples in the U, F, and FU groups were significantly reduced, and the free water content was significantly increased compared with the untreated group. This was in agreement with the moisture ratio data of the material before drying in Fig. 2. This indicated that ultrasonic or freezing pretreatment damaged to the cell wall structure of the material and changed the permeability of the cell membrane, making the water with low freedom and relatively stable state transform into the water with high freedom and unstable state. This explained that FU pretreatment significantly accelerated the drying speed by enhancing free water diffusion in and out the cells.

The content of bound water and free water determines the drying efficiency and drying degree in a way. Fig. 5(B) showed that most free and semi-bound water was removed from the dried strawberry slices after IHAID drying, and the relative peak area decreased significantly. The untreated group indicated the most significant proportion of bound water, yet the FU group exhibited the lowest proportion of bound water.

The MRI images of samples after different pretreatments and drying were presented in Fig. 5(C), which more vividly reflected the moisture changes of samples. There is a significant positive correlation between the water content of the sample and the signal intensity of the MRI, and the red in the image indicated signal strength with high water content. The strawberries in the control group contain a large amount of water and have uneven distribution of water, showing a central position higher than the edge position. However, the water diffused towards the edge of the strawberry slices and became more evenly distributed after U, F, or FU treatments. The colour discontinuity in the colour diagram also confirmed the damage to the microstructure of strawberries caused by pretreatment in Fig. 2. The edge of the dried strawberry slices only has a weak signal, indicating that the moisture content of each group of dry samples was very low, and all reached the drying end. In general, various pretreatment methods have significant effects (*p* < 0.05) on the water state and distribution of strawberry samples.

### Texture changes of strawberry slices under different pretreatments

Texture helps people understand the sensory characteristics of food taste through mechanical analysis. Hardness and chewiness are usually used to reflect the structural changes of food and the degree of cell damage. Compared to the untreated group, the hardness of fresh strawberry slices after U, F, and FU treatment decreased by 52.6 %, 42.4 % and 71.6 %, respectively. The chewiness of different pre-treated strawberry slices also showed a similar trend. Importantly, strawberry slices after FU pretreatment had the lowest hardness and chewiness (Table 1). Differences in textural properties are often due to changes in cell walls and membranes caused by thermal or pressure effects (Ai et al., 2022). However, the texture differences of strawberry slices in this study are mainly caused by the cavitation effect of ultrasound and ice crystals produced by freezing, which damage the cell walls and cell membranes and change the expansion pressure within the cells. This is also consistent with the results of Liu et al. (2021) who further demonstrated the effects of ultrasound and freezing on the cell walls and cell membranes of *Platycodon grandiflorum* slices through optical microscopy imaging.

## Conclusion

The effects of U, F, and FU pretreatment on drying kinetics, colour, phytochemical composition, antioxidant capacity, microstructure, texture and water status of IHAID dried strawberry slices were investigated and compared with the unpretreated control group. FU pretreatment significantly reduced the drying time and effectively improved the drying efficiency compared with other pretreatment methods. FU pretreatment significantly decreased the drying time by 50 % compared to the untreated group. The mechanism for promoting drying is that FU pretreatment would change the internal structure of the material (causing many irregular channels and small cracks inside the sample) and moisture status (converting bound water in the tissue into free water). Colour analysis showed that FU pretreated dried strawberry products showed a more attractive red colour and a* value was higher. In addition, FU pretreatment had a positive impact on the total anthocyanin content (TAC), total phenolic content (TPC), and vitamin C (VC) content, antioxidant activity of strawberry samples, although the chewiness was significantly reduced. U and FU pretreatments could better retain the volatile aroma in strawberries compared with the control group. In conclusion, these findings provide important information for an in-depth understanding of the effects of FU on the drying characteristics and physicochemical properties of strawberry slices and demonstrate that FU is a very promising non-thermal pretreatment technology for strawberry slices. In future research, we will further optimize the FU process and combine it with other pre-treatment methods to improve the texture and taste of strawberry slices, and further promote the application of FU technology for the industrial production of high value-added dried strawberries.

## CRediT authorship contribution statement

**Da-Long Jiang:** Writing – original draft, Investigation, Funding acquisition, Conceptualization. **Qing-Hui Wang:** Writing – review & editing, Resources, Funding acquisition, Conceptualization. **Chu Huang:** Resources, Methodology. **Parag Prakash Sutar:** Writing – review & editing. **Ya-Wen Lin:** Resources, Methodology. **Samuel Ariyo Okaiyeto:** Writing – review & editing. **Zi-Fan Lin:** Writing – original draft. **Yun-Tian Wu:** Resources. **Wen-Ming Ma:** Writing – review & editing. **Hong-Wei Xiao:** Writing – review & editing, Supervision, Resources, Funding acquisition, Conceptualization.

## Declaration of competing interest

The authors declare that they have no known competing financial interests or personal relationships that could have appeared to influence the work reported in this paper.

## Data Availability

Data will be made available on request.
